# Prognostic significance of BRAF and NRAS mutations in melanoma: a German study from routine care

**DOI:** 10.1186/s12885-017-3529-5

**Published:** 2017-08-10

**Authors:** Markus V. Heppt, Timo Siepmann, Jutta Engel, Gabriele Schubert-Fritschle, Renate Eckel, Laura Mirlach, Thomas Kirchner, Andreas Jung, Anja Gesierich, Thomas Ruzicka, Michael J. Flaig, Carola Berking

**Affiliations:** 1 Department of Dermatology and Allergy, University Hospital of Munich (LMU), Frauenlobstr. 9-11, 80337 Munich, Germany; 2 Division of Health Care Sciences, Center for Clinical Research and Management Education, Dresden International University, Freiberger Str. 37, 01067 Dresden, Germany; 3 Munich Cancer Registry (MCR) of the Munich Tumor Centre (TZM), Department of Medical Information Processing, Biometry and Epidemiology (IBE), University Hospital of Munich, Ludwig-Maximilian-University (LMU), Marchioninistr. 15, 81377 Munich, Germany; 4 Department of Pathology, University of Munich (LMU), Thalkirchner Str. 36, 80337 Munich, Germany; 5 Department of Dermatology, University Hospital Würzburg, Josef-Schneider-Str. 2, 97080 Würzburg, Germany; 6 DKTK (German Cancer Consortium), DKFZ (German Cancer Research Centre), Im Neuenheimer Feld 280, 69120 Heidelberg, Germany

**Keywords:** Melanoma, BRAF, NRAS, BRAF inhibitor, MEK inhibitor, Immune checkpoint blockade, Nodal relapse, Overall survival, Survival analysis, Disease progression

## Abstract

**Background:**

Hotspot mutations of the oncogenes BRAF and NRAS are the most common genetic alterations in cutaneous melanoma. Specific inhibitors of BRAF and MEK have shown significant survival benefits in large phase III trials. However, the prognostic significance of BRAF and NRAS mutations outside of clinical trials remains unclear.

**Methods:**

The mutational status of BRAF (exon 15) and NRAS (exon 2 and 3) was determined in melanoma samples of 217 patients with pyrosequencing and Sanger sequencing. The genotypes were correlated with clinical outcomes and pathologic features of the primary tumors. Time to disease progression was calculated with the cumulative incidence function. Survival analyses were performed with Kaplan-Meier estimates and Cox proportional hazards regression analysis. Relative survival was calculated with the Ederer-II method. Treatment with BRAF and MEK inhibitors and immune checkpoint blockade (ICB) was allowed.

**Results:**

Mutations in BRAF and NRAS were identified in 40.1 and 24.4% of cases, respectively. Concurrent mutations in both genes were detected in further 2.3%. The remaining 33.2% were wild type for the investigated exons (WT). BRAF mutations were significantly associated with younger age at first diagnosis (*p* < 0.001) and truncal localization of the culprit primary (*p* = 0.002). The nodular subtype was most common in the NRAS cohort. In addition, NRAS-mutant melanoma patients showed a higher frequency of nodal relapse (*p* = 0.013) and development of metastatic disease (*p* = 0.021). The time to loco-regional nodal relapse was shortest in NRAS-mutant melanoma (*p* = 0.002). Presence of NRAS mutation was an independent risk factor for disease progression in multivariate analysis (HR 2.01; 95% CI 1.02 – 3.98). BRAF-mutant melanoma patients showed a tendency for better overall and relative survival. Genotype was not a consistent risk factor in multivariate analysis. Instead, positive sentinel lymph node status (HR 2.65; 95% CI 1.15 – 6.10) and treatment with ICB in stage IV disease (HR 0.17; 95% CI 0.06–0.48) were significant multivariate risk factors.

**Conclusions:**

NRAS-mutant tumors tended to behave more aggressively particularly in early stages of the disease in this high-risk melanoma population. Treatment with immune checkpoint blockade improved survival in stage IV disease in a real-world setting.

**Electronic supplementary material:**

The online version of this article (doi:10.1186/s12885-017-3529-5) contains supplementary material, which is available to authorized users.

## Background

Melanoma is a malignancy originating from melanocytes of the skin with a high propensity to metastasize. Activating mutations of the oncogenes BRAF and NRAS lead to constitutive signaling of the mitogen-activated protein kinase (MAPK) pathway and thereby enhance tumor growth and promote disease progression [1, 2]. Genetic alterations in both genes can be detected in approximately 40 and 20% of cases, respectively [1]. Although specific tyrosine kinase inhibitors targeting BRAF (BRAFi) and MEK (MEKi) are currently available for metastatic melanoma, the overall biologic and prognostic significance of BRAF and NRAS mutations remains unclear and conflicting evidence exists.

In melanoma, the most common mutation of BRAF is a substitution from valine (V) to glutamic acid (E) in codon 600 of exon 15 (V600E). This particular alteration was previously associated with younger age at initial diagnosis, little chronic UV damage, truncal localization of the primary tumor, and a high total body nevus count [3–7]. Nevertheless, the significance for the further course of the disease such as distant metastasis-free or overall survival (OS) is less clear. One study proposed that mutant BRAF had no impact on the disease-free interval from diagnosis of the culprit tumor to first distant metastasis. However, the median survival in stage IV disease was shorter in patients with BRAF-mutant melanoma and not treated with a BRAF inhibitor than in the wild type (WT) situation [8, 9]. These findings are opposed to other analyses in which patients not treated with BRAF inhibition showed similar survival curves to WT patients [10, 11].

The NRAS gene is most frequently mutated at hotspots in exon 2 (codons 12 and 13) and exon 3 (codon 61) [12]. Mutations of NRAS were previously associated with nodular subtype of the primary tumor and localization in sun-damaged skin [13]. As is the case for BRAF, studies investigating the prognostic relevance of NRAS have revealed discordant results. One of the first examinations dealing with this question identified NRAS as an independent factor indicative of favorable OS [14]. Others have found that NRAS mutation status predicted shorter survival after the diagnosis of stage IV disease [12, 15]. In contrast, no differences in OS after diagnosis of distant metastases were detected in several other investigations [11, 16–18].

To further elucidate the prognostic impact of mutations in the BRAF and NRAS genes, we analyzed the genotype of 217 patients with melanoma and retrospectively correlated the mutation status to primary tumor and clinical data. To account for a real-life situation and respect potential survival benefits achieved with novel therapy options, patients receiving targeted therapy with kinase inhibitors and immune checkpoint blockade (ICB) with ipilimumab, nivolumab, or pembrolizumab were included in our analysis.

## Methods

### Study cohort and data collection

A total of 217 melanoma patients with available information on the mutational status of the oncogenes BRAF and NRAS were included. Clinical and histologic data were retrieved retrospectively from the routine patient records of the dermato-oncology unit of the University Hospital of Munich (LMU) and from the Munich Cancer Registry database (MCR). The MCR is a population-based prospective cancer registry with standardized case report forms for patient characteristics, TNM stage, therapy, follow-up, disease progression, and vital status [19–22].

Cases with in situ melanoma, lentigo maligna, unknown primaries and uveal melanoma were excluded. All patients were classified according to the cutaneous melanoma staging guidelines of the American Joint Committee on Cancer (AJCC) as stated in 2009 [23]. The data were merged to a central database prior to statistical analyses and comprised: demographics, performance and result of sentinel lymph node biopsy (SLNB), TNM stage, AJCC stage, therapies, and coincidence of other malignancies. The primary tumor information was ascertained by the original pathology report and provided information on tumor site, histologic type, Breslow’s depth, Clark’s level, ulceration, and mitotic activity. Mitotic activity was determined by the presence of ≥ 1 mitotic figure. If multiple primary melanomas were present in one patient, the tumor which was considered responsible for disease progression (culprit tumor) was determined with a previously described algorithm [11, 24, 25].

### Tumor samples and mutation analyses

Mutations were tested preferentially in distant or lymph node metastases as heterogeneity of the mutational status between metastatic and primary tumor sites have been reported and as BRAF mutations have predictive value for the use of BRAFi and MEKi in metastatic disease [26, 27]. If no such samples were available, analyses were performed in primaries. Areas of tumor tissue were identified from 10 μm-thick formalin-fixed, paraffin-embedded sections. To enrich for a content of > 75%, tumors were manually micro-dissected with an ultra-thin cannula. DNA was isolated with extraction buffer (Tris-HCl pH 7.4 0.1 M, EDTA 0.5 mM, Tween 20 0.5% in distilled water) after proteinase K hydrolysis for 16 h (Thermo Fisher Scientific, Darmstadt, Germany). Samples were amplified using polymerase chain reactions (PCR) covering BRAF exon 15 (Codon 600), NRAS exon 2 (codon 12, 13), and NRAS exon 3 (codon 61) using specific primers (Additional file 1: Tables S1 and S2). PCR products were subsequently subjected to pyrosequencing with the PyroMark Q24 System (Qiagen, Hilden, Germany) together with specific primers (Additional file 1: Table S2). If sequencing results were unclear or if mutations were detected in both genes, pyrosequencing was followed by additional Sanger sequencing to confirm the mutational status which was performed by MWG Operon (Ebersberg, Germany).

### Endpoints

During follow-up, we defined three distinct types of disease progression: (i) local recurrence, (ii) regional lymph node metastasis or lymph node recurrence for patients who initially presented with nodal disease, and (iii) formation of distant metastasis. For the purpose of analysis, patients were allowed to have more than one type of progression, if the progression events developed successively and if they were evident in timely distinct staging procedures (i.e., lymph node relapse followed by metastatic disease). If more than one progression type occurred at the same time, the event of higher prognostic value was used for the analysis (distant metastasis > lymph node metastasis > local recurrence).

Time to progression (TTP) was determined as time from melanoma diagnosis to first disease progression and documented separately for each type of progression. For time to event analyses, local recurrence and regional lymph node metastasis were analyzed as joint category due to the low lumber of local relapses (loco-regional). OS was defined as the time from the initial diagnosis of melanoma to disease-specific death or to the date of the last documented contact which was used as censored observation. Post-progression survival (PPS) started after the first disease progression and was recorded until death due to any cause occurred. The vital status was recorded and updated regularly by the treating oncologists and validated by death certificates issued by municipal registration offices.

### Statistical analyses

Before inferential analyses were performed, data were analyzed with descriptive measures for central tendencies and variation. Frequency distribution tables were generated for each parameter of interest. Associations with mutation types were analyzed for the three genotypes BRAF-mutant, NRAS-mutant, and WT for both genes. Chi-square and Kruskal-Wallis tests were applied to assess differences of categorical and ordinal data, respectively.

TTP was calculated with the cumulative incidence function according to Kalbfleisch and Prentice in which death was considered a competing risk. The respective curves were compared for significance with the Gray’s test. OS and PPS were computed with the product limit (Kaplan-Meier) method for censored failure time data assuming proportional hazards. Survival curves were compared with the log-rank test. Relative survival (RS) was defined as ratio of the observed to expected survival of a matched melanoma-free population and calculated with the Ederer-II method. Thus, the relative survival represented an accurate and objective estimate of tumor-specific survival (net melanoma survival) and was adjusted for age-dependent mortality [28].

Univariate hazard ratios (HR), 95% confidence intervals (95% CI), and *p*-values for OS were obtained with Cox proportional hazard regression modelling. Associations between independent covariates and OS were calculated with multivariate Cox regression. Associations between risk factors of interest and TTP were assessed in a multivariate risk model where death was considered a competing risk. The reference parameters for Cox regression and competing risk models were age < 50 years, male sex, Breslow’s thickness < 1.00 mm, negative nodal status, WT for BRAF and NRAS, non-nodular subtype, and no systemic therapy. Two-sided *p*-values were calculated in all cases. The significance level for contingency tables was adjusted for multiple hypothesis testing by a family-wise Bonferroni correction, testing each individual hypothesis at a significance level of *p* = 0.05/m with m being the number of comparisons made in the respective table. All analyses were performed with SAS programming package version 9.1 (SAS Institute Inc., Cary, NC, USA).

## Results

### Mutation frequencies of BRAF and NRAS

A total of 217 patients with available information on their BRAF and NRAS genotype status were included. Mutations in either one gene were detected in tumors from 140 patients (64.5%). BRAF but not NRAS was mutated in 87 cases (40.1%), while 53 patients (24.4%) showed NRAS mutations. Genetic alterations in both genes were present in 5 patients (2.3%). Seventy-two patients (33.2%) showed no mutation in BRAF exon 15 or NRAS exon 2 or 3. They were further referred to as WT. The mutation status was assessed in two or more melanoma lesions in 23 patients (10.6%) with concordant genotypes in all cases.

Of patients with BRAF mutations, 63 (72.4%) displayed V600E, 15 (17.2%) V600K, and 4 (4.6%) V600R mutations. Non-V600 mutations of BRAF were present in 5 cases (2.3%). Of patients with NRAS mutations, the majority showed alterations for glutamine at position 61 with Q61K (*n* = 22, 41.5%) or Q61R (*n* = 19, 8.8%) being the most common substitutions. Concurrent mutations of BRAF and NRAS were identified in 5 patients (2.3%), two of whom showed non-V600 mutations in the BRAF gene (Table 1).

**Table 1:** Frequencies and types of BRAF and NRAS mutations

Mutation	Number of patients (n, %)	% of subpopulation
BRAF	87 (40.1)	BRAF (*n* = 87)
V600E	63 (29.0)	72.4
V600K	15 (6.9)	17.2
V600R	4 (1.8)	4.6
K601E	2 (0.9)	2.3
L597S	1 (0.5)	1.1
V600E, K601E	1 (0.5)	1.1
A598A, R603*	1 (0.5)	1.1
NRAS	53 (24.4)	NRAS (*n* = 53)
Q61K	22 (10.1)	41.5
Q61R	19 (8.8)	35.8
Q61L	3 (1.4)	5.7
Q61V	1 (0.5)	1.9
Q61H	1 (0.5)	1.9
A59D	2 (0.9)	3.8
G12D	1 (0.5)	1.9
G13R	2 (0.9)	3.8
BRAF + NRAS	5 (2.3)	BRAF + NRAS (*n* = 5)
V600E, Q61K	1 (0.5)	20.0
V600E, Q61L	1 (0.5)	20.0
V600E, G12S	1 (0.5)	20.0
K601E, Q61L	1 (0.5)	20.0
L584F, Q61K	1 (0.5)	20.0

Mutation frequencies of BRAF (exon 15) and NRAS (exon 2 and 3) are indicated in total numbers (n) and percentages (%). The codons which are affected by the genetic alterations (left column) are indicated by Arabic numbers. The amino acids are indicated by single letter codes with terminator/end codes indicated by an asterisk (*)

Associations with clinical features, characteristics of the primary culprit tumor, and progression and survival data were assessed based on the mutation status of BRAF and NRAS, revealing the three cohorts BRAF-mutant, NRAS-mutant and WT. Because of the small number (*n* = 5) and the unclear significance of concurrent BRAF and NRAS mutations, this group was precluded from further analysis.

### Clinical and pathologic features at primary diagnosis

The diagnoses of the culprit tumors were made between 1970 and 2014. Patients with BRAF-mutant melanoma were significantly younger than those with NRAS mutations or WT patients (median 56 versus 66 and 67 years, respectively; *p* < 0.001). Based on the original pathology reports, all patients were staged according to the staging system of the AJCC from 2009 [23]. Most (95.9%) patients presented with local or nodal disease with no major differences of the initial disease stage between the genotypes (Table 2). Eight patients (4.2%) were diagnosed with metastatic (stage IV) disease. Coincident malignancies were significantly more common in NRAS-mutant, but not in BRAF-mutant nor WT patients (*p* = 0.003).

**Table 2:** Clinical characteristics at primary diagnosis of the culprit tumor

	BRAF *n* = 87 (100.0)	NRAS *n* = 53 (100.0)	Wild type *n* = 72 (100.0)	Total *n* = 212^a^ (100.0)	*p*-value
Gender					
Male	44 (50.6)	29 (54.7)	30 (41.7)	103 (48.6)	0.314
Female	43 (49.4)	24 (45.3)	42 (58.3)	109 (51.4)	
Age					
median (years)	56	66	67	64	0.001*
IQR (years)	44 – 67	54.5 – 76	59 – 75	50 – 72	
< 50	31 (35.6)	9 (17.0)	12 (16.7)	52 (24.5)	0.001*
50 – 59	19 (21.8)	10 (18.9)	6 (8.3)	35 (16.5)	
60 – 69	22 (25.3)	13 (24.5)	20 (27.8)	55 (25.9)	
≥ 70	15 (17.2)	21 (39.6)	34 (47.2)	70 (47.2)	
SLNB^b^					
not performed	42 (48.3)	24 (45.3)	24 (33.3)	90 (43.9)	0.077
performed	41 (49.4)	26 (52.0)	48 (66.7)	115 (56.1)	
SLN positive	23 (26.4)	14 (26.4)	20 (27.8)	57 (26.9)	0.352
SLN negative	18 (20.7)	12 (22.6)	28 (38.9)	58 (27.4)	
AJCC stage^c^					
I	27 (35.5)	14 (29.2)	14 (21.2)	55 (29.0)	0.199
II	18 (23.7)	16 (33.3)	29 (43.9)	63 (33.2)	
III	29 (38.2)	15 (31.3)	20 (30.3)	64 (33.7)	
IV	2 (2.6)	3 (6.3)	3 (4.6)	8 (4.2)	
N status (TNM)^d^					
N0	46 (60.5)	31 (64.6)	43 (64.2)	120 (62.8)	0.980
N1	15 (19.7)	10 (20.8)	14 (20.9)	39 (20.4)	
N2	9 (11.8)	4 (8.3)	7 (10.5)	20 (10.5)	
N3	6 (7.9)	3 (6.3)	3 (4.5)	12 (6.3)	
M status (TNM)					
M0	85 (97.7)	50 (94.3)	69 (95.8)	204 (96.2)	0.585
M1	2 (2.3)	3 (5.7)	3 (4.2)	8 (3.8)	
Other malignancies^e^					
no	75 (91.5)	35 (68.6)	57 (82.6)	167 (82.7)	0.003*
yes	7 (8.5)	16 (31.4)	12 (17.4)	35 (17.3)	

Baseline demographic and clinical characteristics are indicated according to genotype. Categorical variables were compared with the Chi-square test and age differences with the non-parametric Kruskal-Wallis test; the significance level was adjusted with the Bonferroni correction with *p* < 0.006 considered significant (*). ^a^Five patients showed mutations in both BRAF and NRAS; ^b^no data on SLNB performance available for *n* = 7; ^c^no definite AJCC staging available for *n* = 22; ^d^unknown N status for *n* = 21; ^e^no information for other malignancies available for *n* = 10; *SLNB* sentinel lymph node biopsy, *IQR* interquartile range, *AJCC* American Joint Committee on Cancer

The mutation status was strongly associated with the anatomic site of the culprit tumor. Six patients (2.9%) had melanoma of mucosal origin. Among patients who had a cutaneous primary, trunk was the most frequent localization in the BRAF- and NRAS-mutant cohorts (45.9% and 38.0%, respectively; *p* = 0.002). The localization was more evenly distributed in the WT group with no obvious predilection sites (Table 3). Nodular melanoma (NM) was the most frequent histologic type in the entire population (*n* = 85, 41.1%). The highest portion of the aggressive NM subtype was observed in NRAS-mutant patients (52.0%). Although the acral lentiginous type was rarely detected, it appeared more common in the WT than in the mutant cohorts (15.3 versus 3.4% for BRAF and 4.0% for NRAS). There were no significant differences between the three cohorts with respect to Breslow’s depth, Clark’s level, or ulceration of the primary tumor. Likewise, there was no significant association of the T status with the genotype. Mitotic activity was more commonly found in primary tumors of NRAS-mutant and WT patients than in those with BRAF mutations (*p* = 0.002).

**Table 3:** Primary tumor characteristics of the cohorts according to genotype

	BRAF *n* = 87 (100.0)	NRAS *n* = 53 (100.0)	Wild type *n* = 72 (100.0)	Total *n* = 212^a^ (100.0)	*p*-value
Localization					
Acral	3 (3.5)	4 (8.0)	14 (19.4)	21 (10.1)	0.002*
Head / neck	14 (16.5)	5 (10.0)	15 (20.8)	34 (16.4)	
Arms	7 (8.2)	10 (20.0)	10 (13.9)	27 (13.0)	
Trunk	39 (45.9)	19 (38.0)	15 (20.8)	73 (35.3)	
Legs	22 (25.9)	10 (20.0)	14 (19.4)	46 (22.2)	
Mucosal	0 (0.0)	2 (4.0)	4 (5.6)	6 (2.9)	
Histologic subtype^a^					
ALM	3 (3.53)	2 (4.0)	11 (15.3)	16 (7.7)	0.021
LMM	2 (2.35)	0 (0.0)	2 (2.78)	4 (1.93)	
NM	32 (37.7)	26 (52.0)	27 (37.5)	85 (41.1)	
SSM	37 (43.5)	14 (28.0)	17 (23.6)	68 (32.9)	
unclassified	11 (12.9)	8 (16.0)	15 (20.8)	34 (16.4)	
Breslow’s index^b^					
≤ 1.00 mm	43 (50.6)	26 (52.0)	32 (45.1)	101 (49.0)	0.241
1.01 – 2.00 mm	21 (24.7)	9 (18.0)	14 (19.7)	44 (21.3)	
2.01 – 4.00 mm	17 (20.0)	8 (16.0)	12 (16.9)	37 (18.0)	
≥ 4.01 mm	4 (4.7)	7 (14.0)	13 (18.3)	24 (11.7)	
Clark level^c^					
II	3 (3.8)	1 (2.3)	4 (6.6)	8 (4.4)	0.094
III	25 (31.3)	9 (20.9)	9 (14.8)	43 (23.4)	
IV	45 (56.3)	30 (69.8)	36 (59.0)	111 (60.3)	
V	7 (8.8)	3 (7.0)	12 (19.7)	22 (12.0)	
Ulzeration^d^					
no	49 (60.5)	23 (47.9)	36 (50.7)	108 (54.0)	0.301
yes	32 (39.5)	25 (52.1)	35 (49.3)	92 (46.0)	
Mitotic activity^e^					
no	15 (37.5)	4 (16.0)	3 (7.0)	22 (20.4)	0.002*
yes	25 (62.5)	21 (84.0)	40 (93.0)	86 (79.6)	

Pathologic features of the primary tumors are indicated according to genotype. The categorical variables were compared with the Chi-square test; the significance level was adjusted with the Bonferroni correction with *p* < 0.008 considered significant (*). Mitotic activity was defined as presence of ≥ 1 mitosis per high power field. ^a^No data on the histologic subtype for *n* = 5; ^b^no data on Breslow’s index for *n* = 6; ^c^no data on Clark level available for *n* = 28; ^d^ulceration unknown for *n* = 12; ^e^missing data for mitotic activity in primary tumors for *n* = 104; *ALM* acral lentiginous melanoma, *LMM* lentigo maligna melanoma, *NM* nodular melanoma, *SSM* superficial spreading melanoma

### Patterns of disease progression

The overall relapse rate (patients with at least one type of recurrence) was 85.8%. Local relapse at the primary tumor site was rare and observed in only 6 patients (2.8%), 4 of whom showed NRAS mutations (Table 4). A relapse of loco-regional nodal disease was observed in 92 patients (43.3%). The formation of distant metastases was detected in 157 patients (77.0%) during the course of the disease. Notably, relapse of nodal disease was significantly more common in the NRAS-mutant cohort than in WT or BRAF-mutant patients, suggesting that this subgroup was at highest risk for disease progression (*p* = 0.013).

**Table 4:** Patterns of disease progression^a^

	BRAF *n* = 87 (100.0)	NRAS *n* = 53 (100.0)	Wild type *n* = 72 (100.0)	total *n* = 212^a^ (100.0)	*p*-value
Local relapse					
no	86 (98.9)	49 (92.5)	71 (98.6)	206 (97.2)	0.057
yes	1 (1.2)	4 (7.6)	1 (1.4)	6 (2.8)	
Relapse of nodal disease (loco-regional)					
no	56 (64.4)	21 (39.6)	43 (59.7)	120 (56.6)	0.013*
yes	31 (35.6)	32 (60.4)	29 (40.3)	92 (43.4)	
Distant metastases^b^					
no	18 (21.2)	6 (12.0)	23 (33.3)	47 (23.0)	0.021
yes	67 (78.8)	44 (88.0)	46 (66.7)	157 (77.0)	

The frequency and types of disease progression are displayed according to the genotype. Patients were allowed to have more than one progression type. Statistical analyses were performed with the Chi-square test; the significance level was adjusted with the Bonferroni correction with *p* < 0.017 considered significant (*). ^a^ *n* = 4 patients had disease progression, but the specific progression type was unknown; ^b^patients initially presenting in stage M1 (IV) were excluded (*n* = 8)

**Fig. 1:**
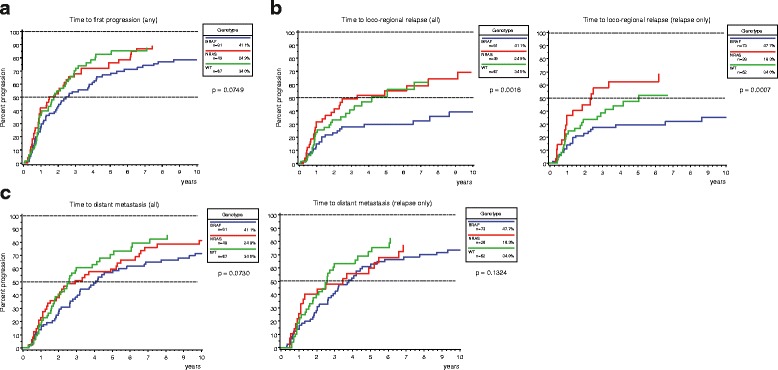
Time from primary diagnosis to first disease progression of any type (**a**), first loco-regional relapse (**b**), and first detection of distant metastases (**c**). Times to the respective events were calculated with the cumulative incidence function and are indicated in years. The BRAF mutant cohort showed the longest time to progression with the median time to first nodal relapse not being reached in the observation period (**b**). In contrast, the NRAS mutant cohort had the shortest time to nodal relapse (**b**), while the median time to progression of any type (**a**) or to the formation of distant metastases (**c**) was almost equal in NRAS mutant and WT melanoma patients. Patients who initially presented with stage IV disease (*n* = 8) were precluded from the analyses shown in (**b**) and (**c**). The right panels of (**b**) and (**c**) indicate progression curves after selection for patients with relapse. The indicated *p*-values were calculated with the Gray’s test

The median time to first disease progression of any type was 2 years (IQR 0.8 – 5.1) for the entire population. According to mutational status, the median TTP was shortest in the NRAS-mutant group (1.5 years, IQR 0.7 – 4.9) and longest for patients whose tumors harbored BRAF mutations (2.4 years, IQR 1 – 7.5). WT patients showed a median TTP of 1.7 years (IQR 0.9 – 3.6; *p* = 0.0749) (Fig. 1a). The median time to loco-regional relapse was shortest in the NRAS group (3.3 years), a finding with high statistical significance (*p* = 0.0016). In contrast, it was not reached by patients who were mutant for BRAF during the observation period (Fig. 1b). In line with these results, the median time from primary diagnosis of the culprit tumor to the detection of distant metastasis was 2.6 and 2.9 years for the WT and NRAS group, respectively, as opposed to 4.1 years in the BRAF cohort (Fig. 1c; *p* = 0.0730). Similar results were obtained when patients with relapse only were selected for TTP analyses (Fig. 1b+c, right panels). Furthermore, NRAS mutational status was an independent risk factor for disease progression in a multivariate risk model where death was considered a competing risk (HR for progression 2.01; 95% CI 1.02 – 3.98; Table 5).

**Table 5:** Multivariate risk model for disease progression

Factor	HR	95% CI	*p*-value
Age			
50 – 59	2.20	1.15 – 4.21	0.017*
60 – 69	1.40	0.68 – 2.89	0.358
≥ 70	1.68	0.79 – 3.59	0.178
Breslow’s index			
1.01 – 2.00 mm	0.93	0.48 – 1.80	0.822
2.01 – 4.00 mm	0.65	0.32 – 1.32	0.229
≥ 4.01 mm	1.43	0.65 – 3.15	0.375
Sex			
female	1.03	0.61 – 1.72	0.921
Histologic subtype			
nodular type	1.29	0.75 – 2.22	0.361
Mutational status			
BRAF	0.75	0.39 – 1.46	0.388
NRAS	2.01	1.02 – 3.98	0.045*
SLNB			
positive	0.95	0.51 – 1.79	0.877

Multivariate model for disease progression where death was considered a competing risk (competing risk model). The references for each factor were age < 50 years, Breslow’s thickness < 1.00 mm, male sex, non-nodular histologic subtype, WT for BRAF and NRAS, and negative SLNB status. Hazard ratios (HR) for progression and 95% confidence intervals (CI) are indicated; *SLNB* sentinel lymph node biopsy, *ICB* immune checkpoint blockade; *p < 0.05

### Post-progression survival

**Fig. 2:**
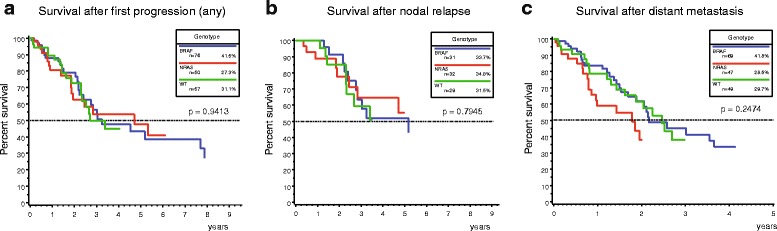
Post-progression survival after first disease progression of any type (**a**), first nodal relapse (**b**), and metastatic disease (**c**). All three genotypes showed similar Kaplan-Meier curves for survival after any disease progression (**a**) and loco-regional nodal recurrence (**b**). Patients who were mutant for NRAS showed a slightly shorter median survival in stage IV disease (1.8 years) compared to patients with BRAF mutant (2.2 years) and WT (2.5 years) melanoma. However, this difference was not significant. The indicated *p*-values were calculated with the log-rank test

The median survival after disease progression was 3.4 years for the entire population (IQR 1.9 – 7.9). Survival time after any disease progression did not show significant differences between all three cohorts (*p* = 0.9413) (Fig. 2a). Median survival after nodal relapse was 5.2 years (IQR 2.3-not reached) and did not show a significant association with genotype (*p* = 0.7945) (Fig. 2b). The median survival in stage IV was 2.1 years (IQR 1.1 – 4.5). Patients who were mutant for NRAS showed the shortest median survival after distant metastases were detected, although this trend was not significant (NRAS 1.8 years, BRAF 2.2 years, WT 2.5 years; *p* = 0.2474).

Thirty-two patients of the entire cohort (14.7%) received targeted therapy with BRAFi or MEKi, 39 (18.0%) received ICB with ipilimumab, pembrolizumab, or nivolumab and 31 (14.3%) investigator-choice chemotherapy. The median survival time of ICB-treated patients was not reached, while it was estimated as 2.1 years for targeted therapy and 1.9 years for chemotherapy. The Kaplan-Meier curves of patients treated with ICB were superior over those treated with targeted therapy or chemotherapy, but this difference was not significant (*p* = 0.0805).

### Overall and relative survival

**Fig. 3:**
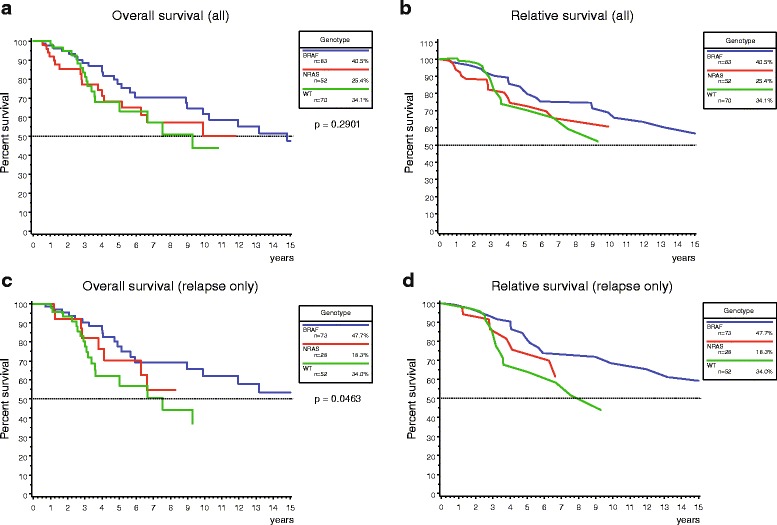
OS and relative survival from diagnosis of the culprit melanoma. **a** + **c** Kaplan-Meier curves are shown for overall survival according to genotype for the entire cohort (**a**) and after selection for patients with relapse (**c**). BRAF-mutant melanoma patients showed a trend towards longer survival compared to NRAS-mutant and WT melanoma patients, which was significant in patients with relapse only. The indicated *p*-value was calculated with the log-rank test. **b** + **d** Relative survival was defined as ratio of the observed survival of the study cohort to the expected survival of a reference population to adjust for cause- and age-specific mortality. Similar results were observed compared to the Kaplan-Meier curves from (**a**) and (**c**)

Median survival time from primary diagnosis to death (OS) was 11.9 years for the entire collective (IQR 4.1-not reached). The median follow-up times were 4.2 years (IQR 1.7 – 6.9) for the BRAF cohort and 3.9 years (IQR 1.5 – 7.1) for the NRAS cohort. WT patients had a shorter follow-up time of 2.8 years (IQR 1.2 – 4.2). To internally validate the dataset, OS was analyzed according to disease stage at primary presentation. Survival curves showed a significant difference from stages I to IV (Additional file 2: Figure S1; *p* = 0.0045). According to genotype, BRAF-mutant individuals showed a trend for longer median OS and relative survival when compared to the NRAS-mutant and WT ones (14.8, 10.2 and 9.3 years, respectively). This difference was statistically significant for patients with relapse only (*p* = 0.0463), but not for the entire cohort (Fig. 3).

Other malignancies were commonly found in NRAS-mutant patients. To determine if non-melanoma cancers influenced survival and caused earlier demise particularly in the NRAS cohort, OS and RS were calculated after exclusion of patients with other malignancies. Indeed, the 5-year OS rate for the NRAS group increased from 68.2 to 74.2% after exclusion of these cases, whereas the other two cohorts were less affected. In contrast, the progression times to loco-regional relapse and to formation of distant metastases were left virtually unaltered by this selection (Additional file 3: Figure S2).

In addition, this survival benefit was not consistent in multivariate analyses with validated prognostic markers of melanoma, as the mutational status of neither BRAF nor NRAS was significantly associated with better OS (BRAF: HR for death 0.46, 95% CI 0.20 – 1.07; NRAS: HR for death 0.70, 95% CI 0.33–1.47; Table 6). Instead, we identified a positive sentinel lymph node status (HR 2.65; 95% CI 1.15–6.10) and treatment with ICB in stage IV disease (HR 0.17; 95% CI 0.06–0.48) as multivariate risk factors.

**Table 6:** Cox regression analyses of validated risk factors for melanoma

	Univariate			Multivariate		
Factor	HR	95% CI	*p*-value	HR	95% CI	*p*-value
Age						
50 – 59	1.53	0.70 – 3.34	0.285	1.53	0.70 – 3.34	0.285
60 – 69	1.62	0.74 – 3.55	0.230	1.62	0.74 – 3.54	0.230
≥ 70	1.35	0.55 – 3.30	0.507	1.35	0.55 – 3.30	0.507
Breslow’s index						
1.01 – 2.00 mm	0.79	0.39 – 1.59	0.502	0.79	0.39 – 1.59	0.502
2.01 – 4.00 mm	0.59	0.27 – 1.29	0.182	0.59	0.27 – 1.29	0.182
≥ 4.01 mm	1.55	0.61 – 3.92	0.356	1.55	0.61–3.92	0.356
Sex						
female	1.72	0.98 – 3.06	0.059	1.72	0.98 – 3.06	0.059
Histologic subtype						
nodular type	1.29	0.55 – 3.02	0.553	1.29	0.55 – 3.02	0.553
Mutational status						
BRAF	0.46	0.20 – 1.07	0.072	0.46	0.20 – 1.07	0.072
NRAS	0.70	0.33 – 1.47	0.346	0.70	0.33 – 1.47	0.346
SLNB						
positive	2.65	1.15 – 6.10	0.022*	2.65	1.15 – 6.10	0.022*
Treatment						
targeted therapy	1.10	0.49 – 2.46	0.821	1.10	0.49 – 2.46	0.821
ICB	0.17	0.06 – 0.48	0.001***	0.17	0.06 – 0.48	0.001***
chemotherapy	0.57	0.25 – 1.29	0.175	0.57	0.25 – 1.29	0.175

Univariate and multivariate analysis on survival from the primary diagnosis (OS) of melanoma. Validated risk factors, the mutational status, and therapies were assessed with Cox proportional hazard regression. The references for each factor were age < 50 years, male sex, Breslow’s thickness < 1.00 mm, negative SLNB status, WT for BRAF and NRAS, non-nodular subtype, and no systemic therapy. Hazard ratios (HR) for death and 95% confidence intervals (CI) are indicated; *SLNB* sentinel lymph node biopsy, *ICB* immune checkpoint blockade; *p < 0.05, ***p < 0.001

## Discussion

In this study, we assessed genotype-phenotype correlations in 217 patients with melanoma. BRAF mutations were identified in 40.1% and NRAS mutations in 24.4%, while no alterations in either gene were found in the remaining 33.2%. Even though the genotype was not a risk factor in multivariate analysis, BRAF-mutant melanoma patients showed a trend towards better overall and relative survival. In contrast, NRAS-mutant patients were more likely to develop early nodal relapse and metastatic disease than the BRAF-mutant or WT cohort suggesting that they were at highest risk for disease progression and ultimately disease-specific mortality.

Regarding characteristics of the primary tumor, trunk was the most common localization in BRAF-mutant patients. The primaries of this cohort showed less mitotic activity than the NRAS and the WT cohorts, in keeping with a prior study [15]. The nodular subtype was more common in BRAF- and NRAS-mutant groups, while there were no major differences regarding other histologic risk factors such as tumor depth or presence of ulceration. These results are in line with previous investigations in which BRAF mutations preferentially occurred in melanomas of skin with little solar elastosis and less commonly affected the head and neck area [3, 7].

Conversely, NRAS mutations had previously been associated with higher Breslow’s thickness and fast growth of the primary tumor [15, 16]. Although this correlation was not confirmed in our collective, NRAS-mutant tumors accounted for 4 out of 6 local relapses at the primary tumor site and loco-regional recurrence and distant metastasis were significantly more commonly observed in NRAS-mutant patients. The time to nodal recurrence was significantly shortest in the NRAS cohort. These results were further confirmed in a multivariate model for disease progression. Even though NRAS mutational status was not a significant risk factor for OS in multivariate analyses, these results imply that NRAS-mutant tumors appear intrinsically more aggressive and are at high risk for disease progression. This trend for shorter relapse-free survival was also supported by other large-scale analyses [12, 15]. However, in stage IV, the survival differences between NRAS-mutant and non-NRAS-mutant patients proposed previously were not statistically significant in our study, even though NRAS-mutant patients showed a tendency towards shorter survival when metastatic lesions were detected [12, 14]. OS defined as time from the first diagnosis of melanoma to death did not show differences between the genotypes in multivariate analyses, suggesting that the impact of NRAS mutations are more evident in earlier disease stages as proposed by Ellerhorst and colleagues [16]. After 5 and 10 years, the BRAF cohort showed a slight survival advantage of 10–15%, yet without significance for the entire cohort. Post-hoc sample size calculations revealed that at least 1239 patients would have been necessary to significantly assess such differences in survival. Thus, our study was likely underpowered. Nevertheless, these findings raise the question on whether NRAS-mutant patients should undergo a more intensive follow-up after primary diagnosis and whether large trials should stratify for NRAS as independent risk factor.

The strengths of this analysis include the reconstruction of the entire interval from diagnosis of the primary culprit tumor to death or last follow-up as censoring event. Based on our data, we conclude that BRAF and NRAS alterations rather affect early disease stages. Thus, it is indispensable to analyze the complete course of the disease and not only focus on stage IV disease where the differences between the genotypes may be less evident. Furthermore, we assessed the prognostic significance of BRAF and NRAS mutations in the presence of novel therapies such as BRAFi and MEKi or ICB which are broadly available nowadays and led to significant survival benefits in phase II and III trials [29, 30]. However, data in the real-world setting is sparse. Most studies proposing that the mutation status is not prognostic in stage IV disease were conducted either before BRAFi and MEKi were accessible outside of trials or precluded patients who were treated with BRAFi and MEKi [11, 31, 32]. To reflect a real-world situation available outside of clinical trials, we believe that is important to take these therapies into consideration.

Our study does have several limitations. First, the cohort was sampled based on availability of tissue as well as of the mutation status, regardless of other demographic and clinical parameters. Although this approach was performed in several other sound reports [3, 12, 16], sampling based on the sole availability of BRAF and NRAS mutational status may bear potential for bias. While it minimizes a potential survivorship bias, it may enrich for patients who underwent surgery or invasive biopsies in stage III or IV disease to obtain tumor material for the mutation analyses. Our collective may not be representative of the target population, because we included patients, in whom molecular testing was clinically indicated due to disease progression. Indeed, the high overall relapse rate of 85.8% demonstrates that our population was skewed towards high-risk melanoma patients. Thus, our results may not be generalizable to all melanoma patients and, in particular, may not be valid for individuals with lower risk disease. To estimate the effect of this bias, we performed time to event analyses including patients with relapse only. The time to loco-regional relapse was less favorable for NRAS-mutant patients and overall as well as relative survival significantly more favorable for BRAF-mutant patients after this selection. These data were largely consistent with our main findings within the entire population. Nevertheless, we cannot exclude that the lack of inclusion of lower risk disease potentially reinforces selection bias.

In addition to a potential sampling bias, further shortcomings of our study include the retrospective design and low number of patients with rare mutations of BRAF and NRAS. The mutation BRAF V600K had previously been associated with older age, head and neck localization of the primary tumor, shorter distant metastasis-free and shorter OS compared to V600E [11, 18, 33]. We identified 15 patients with V600K-mutant melanoma accounting for 6.9 and 17.2% of the entire and BRAF-mutant population, respectively. This proportion is somewhat lower than reported before and precluded further subgroup analyses due to the small number of cases. Non-V600 mutations of BRAF were reported in 7 (3.2%) patients in our study, two of whom had a concomitant mutation in NRAS. More specifically, the mutations K601E and L597S were identified in 5 (2.3%) patients. The small number limited further statistical analyses and conclusions on this particular subpopulation. Others have provided evidence that the alterations K601E and L597 show distinct clinicopathologic features from general V600E/K mutations. The so far largest series on these mutations suggested that tumors harboring K601E mutations are rather similar to tumors with V600E mutations, whereas those with L597 mutations were akin to BRAF WT tumors [34]. However, data regarding treatment responses to BRAFi and the clinical prognosis is sparse due to the paucity of these mutations and exclusion from major trials [34, 35].

## Conclusions

We provide important insights into the significance of BRAF and NRAS mutations in a real-world setting when BRAFi, MEKi, and ICB were largely available. NRAS-mutant melanoma patients showed a significantly higher portion of nodal relapse and the shortest time to loco-regional nodal relapse. Positive SLNB was a significant risk factor and treatment with ICB in stage IV a significant protective factor for OS in multivariate analysis. Even though the mutational status was not a consistent risk factor for OS in multivariate analysis, our results suggest that NRAS-mutant tumors tend to behave more aggressively than their BRAF-mutant and WT counterparts in a high-risk melanoma population.

## Additional files

Additional file 1: **Table S1.** Primer sequences for PCR of the genes BRAF (exon 15), NRAS (exon 2), and NRAS (exon 3). **Table S2.** Probes for pyrosequencing of the genes BRAF (exon 15), NRAS (exon 2), and NRAS (exon 3). (DOCX 14 kb)

Additional file 2: Figure S1. Kaplan-Meier estimates for overall survival according to disease stage. Patients were staged according to the current staging system of AJCC from 2009 at primary diagnosis to internally validate the dataset. The survival curves showed a clear and significant stratification from stage I to stage IV. The indicated *p*-value was calculated with the log-rank test. (PDF 287 kb)

Additional file 3: Figure S2. Time to event analyses after patients with other malignancies than melanoma were excluded (*n* = 179). The times to loco-regional relapse and metastatic disease were assessed with the cumulative incidence function. Indicated *p*-values were calculated with the Gray’s test. Overall and relative survival were computed with the Kaplan-Meier and Ederer-II method, respectively. The *p*-value for overall survival was calculated with the log-rank test. (PDF 358 kb)

## Abbreviations

AJCC: American Joint Committee on Cancer
ALM: Acral lentiginous melanoma
BRAFi: BRAF inhibitor
CI: Confidence interval
HR: Hazard ratio
ICB: Immune checkpoint blockade
LMM: Lentigo maligna melanoma
MAPK: Mitogen-activated protein kinase
MCR: Munich Cancer Registry
MEKi: MEK inhibitor
NM: Nodular melanoma
OS: Overall survival
PCR: Polymerase chain reaction
PPS: Post-progression survival
SLNB: Sentinel lymph node biopsy
SSM: Superficial spreading melanoma
TTP: Time to progression
WT: Wild type
