# Supplementary material for: Prognostic significance of BRAF and NRAS mutations in melanoma: a German study from routine care
Source: BMC Cancer. 2017 Aug 10;17:536. doi: 10.1186/s12885-017-3529-5 (PMC5553744; doi:10.1186/s12885-017-3529-5)
Supplement: Supplementary file 1 — Table S1. Primer sequences for PCR of the genes BRAF (exon 15), NRAS (exon 2), and NRAS (exon 3). Table S2. Probes for pyrosequencing of the genes BRAF (exon 15), NRAS (exon 2), and NRAS (exon 3). (DOCX 14 kb) [file 12885_2017_3529_MOESM1_ESM.docx]

**Supplementary table 1**

| **Gene** | **Exon** | **Primer sequence** |
| --- | --- | --- |
| BRAF | Exon 15 | tgaagacctcacagtaaaaatagg (forward)  BIO~tccagacaactgttcaaactgat (reverse) |
| NRAS | Exon 2 | caacaggttcttgctggtgt (forward)  BIO~cctcacctctatggtgggat (reverse) |
|  | Exon 3 | gattcttacagaaaacaagtg (forward)  BIO~atgacttgctattattgatgg (reverse) |

BIO = biotinylated.

**Supplementary table 2**

| **Gene** | **Exon** | **Probe sequence** |
| --- | --- | --- |
| BRAF | Exon 15 | gtaaaaataggtgattttgg |
| NRAS | Exon 2 | caaactggtggtggttggag |
|  | Exon 3 | gttggacatactggatacagct |
