# Supplementary figures and images for: Prognostic significance of BRAF and NRAS mutations in melanoma: a German study from routine care
Source: BMC Cancer. 2017 Aug 10;17:536. doi: 10.1186/s12885-017-3529-5 (PMC5553744; doi:10.1186/s12885-017-3529-5)

# Overall survival

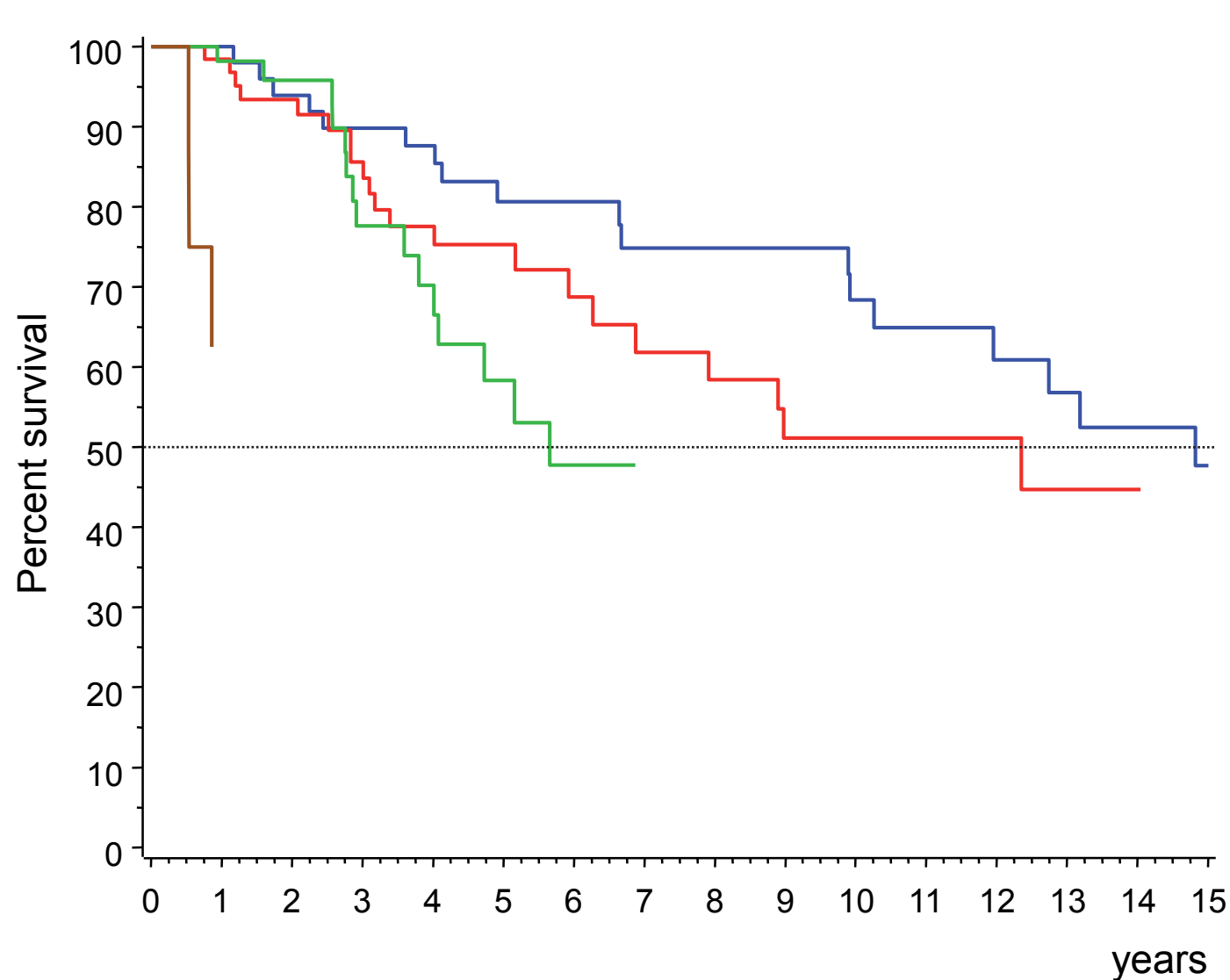

| AJCC stage |      |       |
|------------|------|-------|
| I          | n=55 | 28.8% |
| II         | n=65 | 34.0% |
| III        | n=63 | 33.0% |
| IV         | n=8  | 4.2%  |

p=0.0045

Supplement: Supplementary file 2 — Kaplan-Meier estimates for overall survival according to disease stage. Patients were staged according to the current staging system of AJCC from 2009 at primary diagnosis to internally validate the dataset. The survival curves showed a clear and significant stratification from stage I to stage IV. The indicated p-value was calculated with the log-rank test. (PDF 287 kb) [file 12885_2017_3529_MOESM2_ESM.pdf]

Time to loco-regional relapse

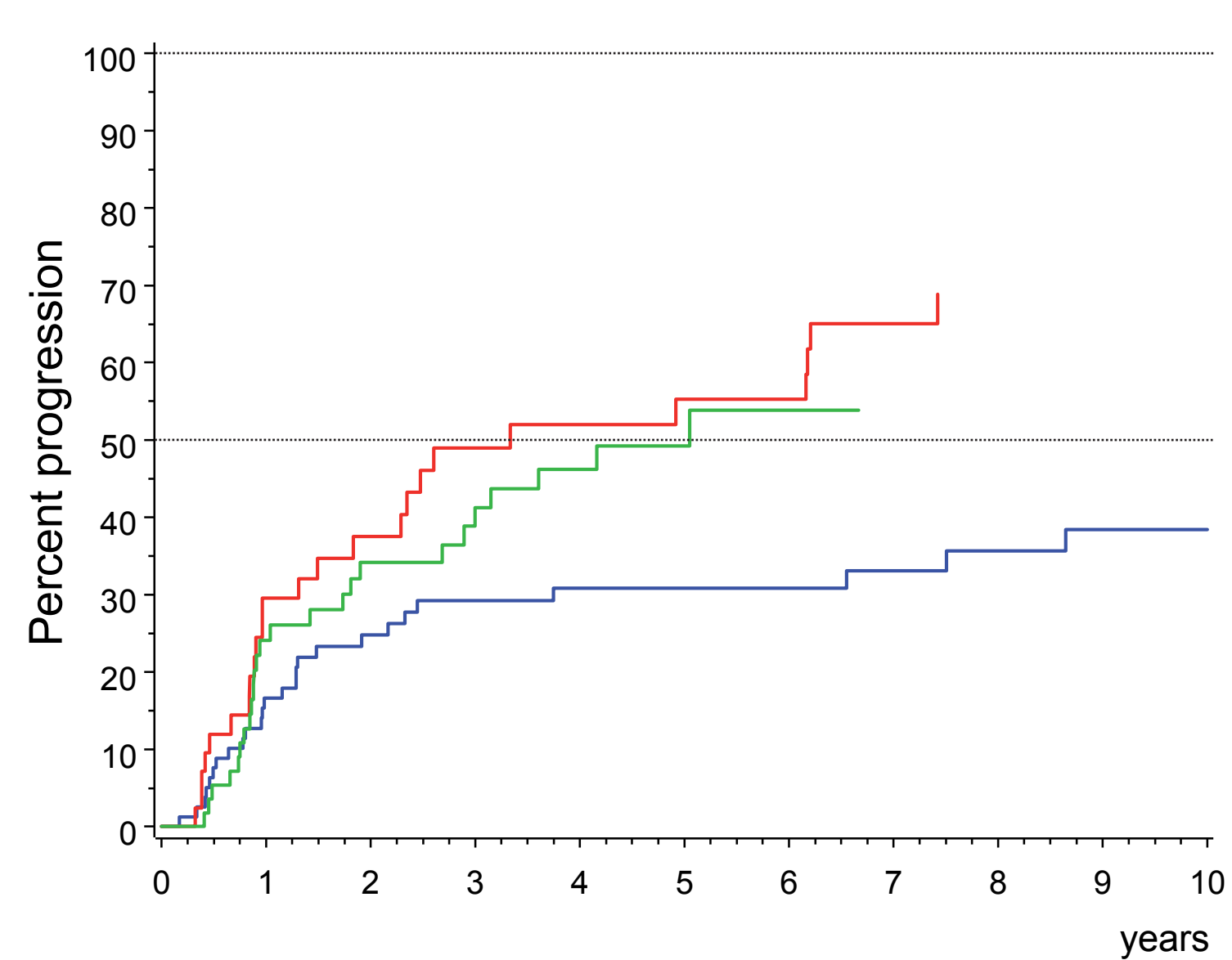

Time to distant metastasis

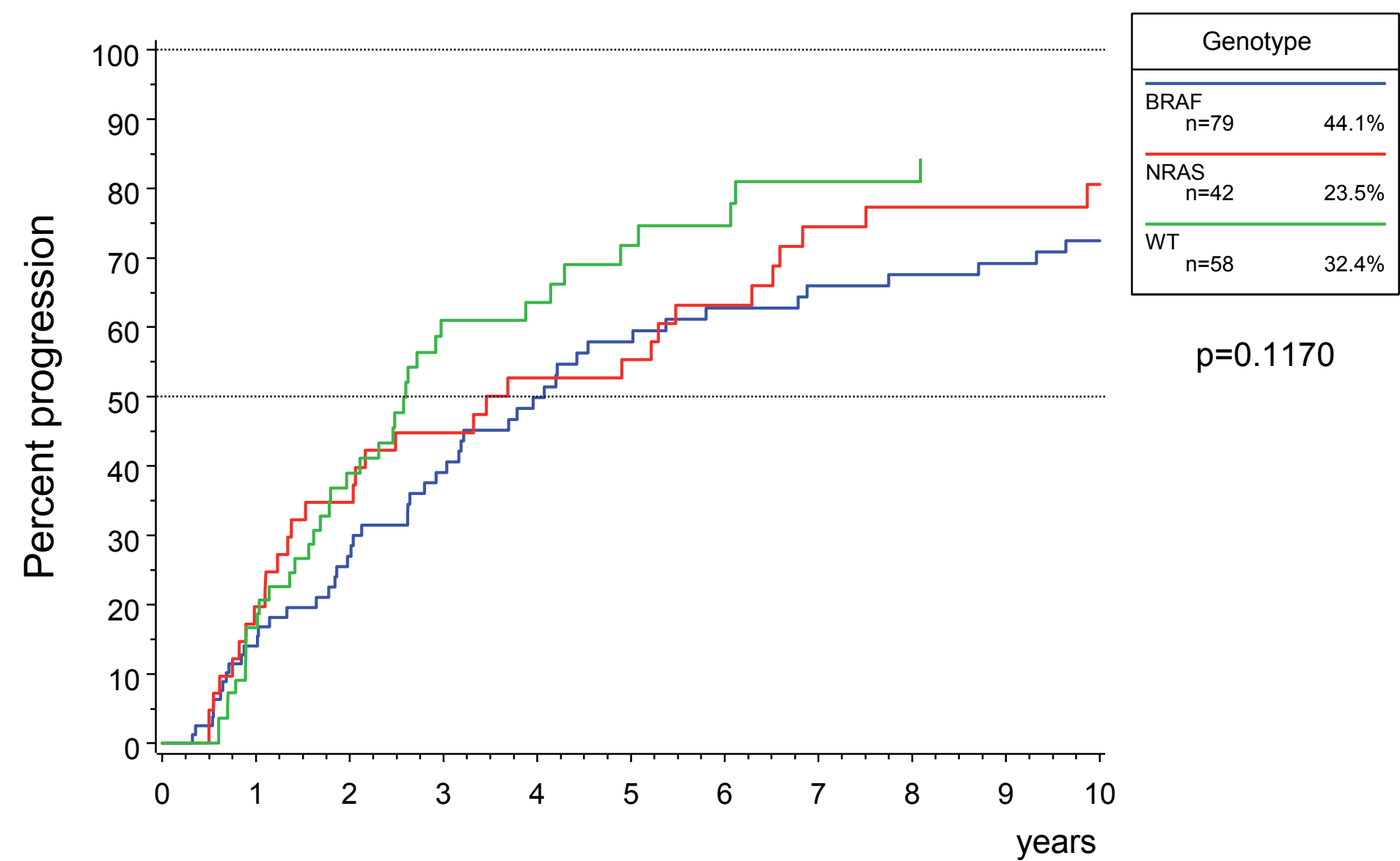

Overall survival

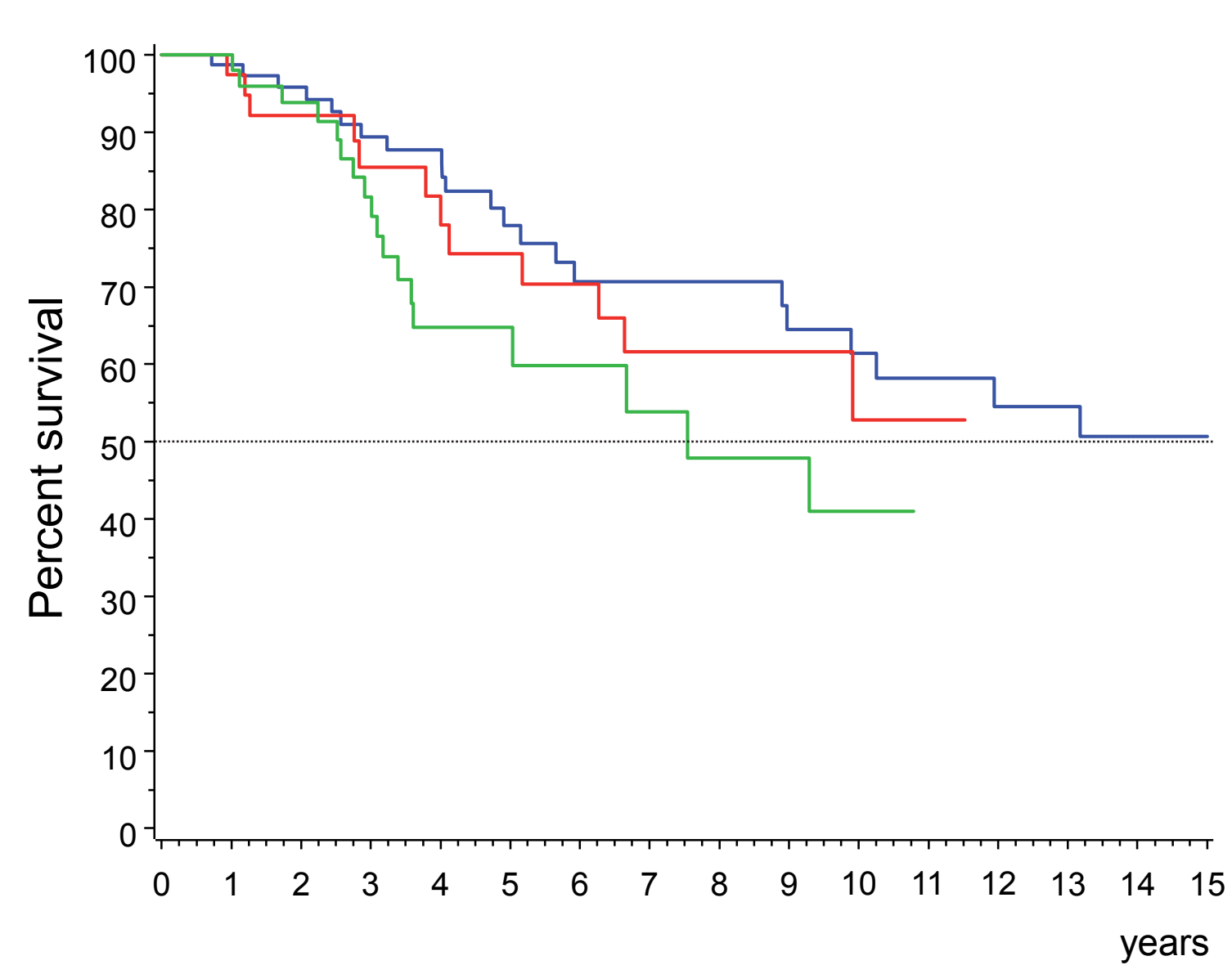

Relative survival

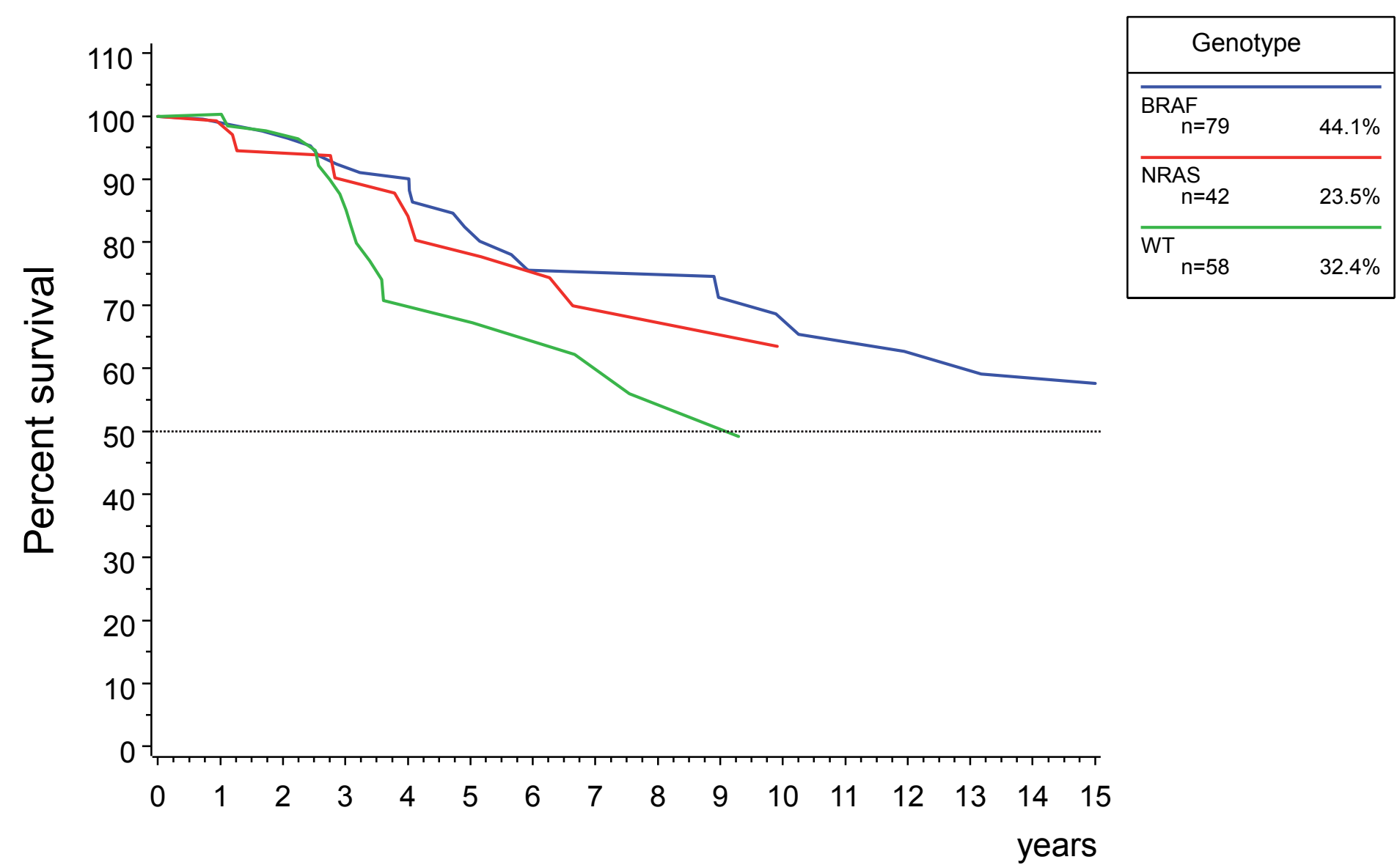

Supplement: Supplementary file 3 — Time to event analyses after patients with other malignancies than melanoma were excluded (n = 179). The times to loco-regional relapse and metastatic disease were assessed with the cumulative incidence function. Indicated p-values were calculated with the Gray’s test. Overall and relative survival were computed with the Kaplan-Meier and Ederer-II method, respectively. The p-value for overall survival was calculated with the log-rank test. (PDF 358 kb) [file 12885_2017_3529_MOESM3_ESM.pdf]
